# COVID-19-related cancellation of elective orthopaedic surgery caused increased pain and psychosocial distress levels

**DOI:** 10.1007/s00167-021-06529-4

**Published:** 2021-03-12

**Authors:** Carolin Knebel, Max Ertl, Ulrich Lenze, Christian Suren, Andreas Dinkel, Michael T. Hirschmann, Ruediger von Eisenhart-Rothe, Florian Pohlig

**Affiliations:** 1 Department of Orthopaedic Surgery, Klinikum Rechts der Isar, Technical University Munich, Ismaninger Strasse 22, 81675 Munich, Germany; 2 Department of Psychosomatic Medicine, Klinikum rechts der Isar, Technical University of Munich, Langerstrasse 3, 81675 Munich, Germany; 3 Department of Orthopaedic Surgery and Traumatology, Kantonsspital Baselland (Bruderholz, Liestal, Laufen), 4101 Bruderholz, Switzerland

**Keywords:** Coronavirus, COVID, COVID-19, SARS-CoV-2, Arthroplasty, Arthroscopy, Pandemic, Orthopedic surgery, Depression, Psychosocial distress

## Abstract

**Purpose:**

Health care systems in most European countries were temporarily restructured to provide as much capacity as possible for the treatment of coronavirus disease 2019 (COVID-19) patients. Subsequently, all elective surgeries had to be cancelled and postponed for months. The aim of the present study was to assess the pretreatment health status before and after COVID-19-related cancellation and the psychosocial distress caused by the cancellation.

**Methods:**

For this study, a questionnaire was developed collecting sociodemographic data and information on health status before and after the cancellation. To assess psychosocial distress, the validated depression module of the Patient Health Questionnaire (PHQ-9), was implemented. PHQ-9-Scores of 10 and above were considered to indicate moderate or severe depressive symptoms. In total, 119 patients whose elective orthopaedic surgery was postponed due to the COVID-19 pandemic were surveyed once at least 8 weeks after the cancellation.

**Results:**

Seventy-seven patients (65%; 34 female, 43 male) completed the questionnaire and were included. The predominant procedures were total knee arthroplasty (TKA), hip arthroscopy and foot and ankle surgery. The mean pain level significantly increased from 5.5 ± 2.2 at the time of the initially scheduled surgery to 6.2 ± 2.5 at the time of the survey (*p* < 0.0001). The pain level before cancellation of the surgery was significantly higher in female patients (*p* = 0.029). An increased analgetic consumption was identified in 46% of all patients. A mean PHQ-9 score of 6.1 ± 4.9 was found after cancellation. PHQ-9 scores of 10 or above were found in 14% of patients, and 8% exhibited scores of 15 points or above. Significantly higher PHQ-9 scores were seen in female patients (*p* = 0.046). No significant differences in PHQ-9 scores were found among age groups, procedures or reasons for cancellation.

**Conclusion:**

Cancellation of elective orthopaedic surgery resulted in pain levels that were significantly higher than when the surgery was scheduled, leading to increased analgesic use. Additionally, significant psychosocial distress due to the cancellation was identified in some patients, particularly middle-aged women. Despite these results, confidence in the national health care system and in the treating orthopaedic surgeons was not affected.

**Level of evidence:**

Level III.

**Supplementary Information:**

The online version contains supplementary material available at 10.1007/s00167-021-06529-4.

## Introduction

On March 11th 2020, the World Health Organization (WHO) declared COVID-19 a pandemic due to the rapid spread around the globe. As a response, health care systems in most European countries were temporarily restructured to provide as much capacity as possible for the treatment of COVID-19 patients. Subsequently, all elective surgeries had to be cancelled and postponed for months [7, 13, 20].

In this context, for many elective orthopaedic procedures, e.g., total hip or knee arthroplasty, it is known that longer waiting times may adversely affect the postoperative outcome [30]. Furthermore, higher levels of pain and loss of physical function can be preoperatively observed [6]. Several studies have also demonstrated that longer waiting times are associated with a poorer pretreatment health status [7, 8, 28]. Although patients with poorer pretreatment health status may benefit more from surgery, Fielden et al. demonstrated that post-treatment health status remained lower than in patients who received treatment more quickly [8].

On the other hand, aspects of mental health often remain underestimated. Psychosocial distress, a term used to describe a variety of psychosocial symptoms, including depression, poor coping, anxiety and somatization, is known to negatively influence postoperative results in patients with musculoskeletal disorders [4, 21]. Approximately 25% of patients undergoing total hip or knee arthroplasty exhibit relevant levels of psychosocial distress resulting in higher postoperative pain levels and poor physical function [21]. In particular, Ayers et al. demonstrated inferior short- and mid-term results in patients with high levels of psychosocial distress undergoing total knee arthroplasty [1]. These results emphasize the importance of mental health for favourable postoperative outcomes in orthopaedic patients.

For the present study it was hypothesized that the COVID-related cancellation and postponement of the scheduled elective orthopaedic surgery lead to (1) higher pretreatment pain levels and (2) psychosocial distress with depressive symptoms.

## Material and methods

The present study was approved by the local ethics committee (IRB 313/20, Technical University of Munich) and informed consent was acquired from every subject prior to inclusion.

All patients (*n* = 119) whose elective orthopaedic surgery at a large university hospital was postponed due to the COVID-19 pandemic between March 12th and April 30th 2020 were included in the present study. Patients were either contacted by email with a link to the online version of the questionnaire or by mail with a paper version of the questionnaire and a prepaid return envelope at least 8 weeks after the cancellation. Detailed information about the purpose of the study and instructions for responding were included. After 7 days, all patients were sent a reminder email or letter. Three weeks after the initial contact, study enrolment was closed.

Seventy-seven completed surveys were collected within the study period, resulting in a response rate of 65%. Almost equal distributions were found regarding gender and age groups (Table 2). The predominant procedures in the study cohort were total knee arthroplasty (TKA) (30%), hip arthroscopy (26%) and foot and ankle surgery (14%). Seventy-nine percent of the procedures were cancelled by the hospital due to national governmental restrictions during the pandemic, whereas 16% of the patients cancelled themselves because of concerns regarding a potential COVID-19 infection. Sixty-one percent of the surveyed patients would have undergone surgery even during the ongoing pandemic if possible. Only 3% of all patients cancelled the surgery to leave capacity for COVID-19 treatment.

### Questionnaire

For the present study, a questionnaire including a general section with sociodemographic and medical information was developed. This section included items on age group, sex, type of elective orthopaedic surgery, subjective urgency for surgical treatment and the reason for cancellation (Supp. 1). The pain level at the time when the operation was scheduled and after the COVID-19-related cancellation of the surgery was assessed by the visual analogue scale (VAS). Furthermore, 12 items regarding personal and occupational restrictions due to the orthopaedic disease and the associated loss of confidence in treating physicians and the health system utilizing a five-level Likert scale were included.

The questionnaire was combined with a previously published self-assessment distress measurement tool, the depression module of the *Patient Health Questionnaire (PHQ)* to assess the level of distress associated with the postponement of the scheduled surgery [18, 19]. With only nine items, the PHQ-9 is only half the length of many other depression measures yet exhibits comparable sensitivity and specificity for a standard cut-off score of 10 or above, as previously described by Kroenke et al. [18]. According to a recent study by Levis et al., specificity can be maximized with a cut-off score of 15 points but at the expense of sensitivity [19]. Thus, the PHQ-9 is a suitable and validated self-assessment measure to accurately screen patients for depressive symptoms. All items of the PHQ-9 assess the occurrence of certain depression symptoms within the past two weeks by means of a four-level scale (never = 0 points, on single days = 1 point, on more than half of all days = 2 points, almost every day = 3 points). The total PHQ-9 score was then interpreted according to Table 1. According to Kroenke et al. and Levis et al., 10 points were considered as the cut-off for psychosocial distress, whereas 15 points or above was interpreted as clinically relevant depression [18, 19] (Table 2).

**Table 1:** PHQ-9 Values and interpretation

Total value	Severity of depression
1–4	Minimal depressive symptoms
5–9	Mild depressive symptoms
10–14	Moderate depressive symptoms
15–27	Severe depressive symptoms

**Table 2:** Sociodemographic data, types of procedures and reasons for cancellation; absolute number of patients is given with percentage in brackets

		Number of patients	PHQ-9		*p* value
			Mean score	SD	
Gender	Female	34 (44%)	7.4	5.3	0.046*
	Male	43 (56%)	5.1	4.4	
Age group	< 30a	5 (6%)	5.6	2.5	
	30-49a	19 (25%)	5.7	5.5	n.s
	50-59a	14 (18%)	7.2	5.6	n.s
	60-69a	16 (21%)	6.7	6.2	n.s
	70-79a	18 (23%)	5.1	3.3	n.s
	> 80a	5 (7%)	7.0	3.1	n.s
Procedure	THA	7 (9%)	7.3	4.9	
	Revision THA	4 (5%)	7.0	7.7	n.s
	TKA	23 (30%)	6.3	4.4	n.s
	Revision TKA	4 (5%)	8.3	6.3	n.s
	Hip arthroscopy	20 (26%)	6.4	5.7	n.s
	Arthroscopic biopsy	4 (5%)	8.8	2.5	n.s
	Foot and ankle surgery	11 (14%)	3.5	3.1	n.s
	Benign tumor resection	4 (5%)	3.0	3.5	n.s
Reason for cancellation	Fear of COVID infection	12 (16%)	4.8	4.6	
	Leaving capacity for COVID patients	2 (3%)	2.0	0.0	n.s
	Cancellation by hospital	61 (79%)	6.3	4.9	n.s
	Personal reasons	2 (3%)	11.0	5.7	n.s
Total		77 (100%)	6.1	4.9	

Mean PHQ-9 scores for each subgroup with standard deviation (SD); *p* value is given for differences in PHQ-9 scores among all groups

*Marks statistically significant differences with *p* ≤ 0.05

### Statistical analysis

Statistical analysis was performed with SPSS Statistics 26 (IBM, Armonk, USA). Descriptive statistics were calculated for all sample characteristics. Mean pain levels before and after cancellation of the surgery were calculated and compared using a t-test for paired samples.

Mean PHQ-9 scores were calculated for each subgroup and compared by two-way ANOVA and a Hochberg and Games-Howell post hoc test. A *p* value of ≤ 0.05 was considered statistically significant.

Sample-size power analysis of *β* = 0.20 and *α* = 0.05 was performed using the prevalence for moderate and severe depression in the general population and the anticipated incidence of depression in the study group. Based on this analysis, a minimum of 67 subjects was needed to power the study adequately.

## Results

The mean pain level significantly increased from 5.5 ± 2.2 at the time of the initially scheduled surgery to 6.2 ± 2.5 at the time of the survey (*p* < 0.0001). Accordingly, 29 patients (38%) declared that they had increased analgesic use. The pain level before cancellation of the surgery was significantly higher in female patients (*p* = 0.029). No significant difference in pain level regarding gender was seen after the cancellation. Patients waiting for resection of a benign musculoskeletal tumour showed significantly lower pain levels before and after the cancellation (*p* = 0.011; *p* = 0.016) than the pain levels associated with all other procedures. Age group and the reason for cancellation had no significant impact on pain level.

No significant loss of confidence in the national health system, the hospital or the orthopaedic surgeon was identified in the present cohort (Table 3). Significant limitations in private (56%) and occupational life (30%) were reported due to cancelled surgery. A vast majority of the surveyed patients (88%) did not reconsider the subjective necessity of the surgery after the cancellation.

**Table 3:** Results of items on confidence in national health system, impact of the disease on personal life and the urgency of the surgery; a five-level Likert scale was applied: “does NOT apply” = 1 point to “does completely apply” = 5 points

Due to the COVID-19 related cancellation of my orthopedic surgery…	Does NOT apply (1)	Does rather NOT apply (2)	Undecided (3)	Does rather apply (4)	Does completely apply (5)	Mean point value	SD
… I lost confidence in the public health system	74%	17%	5%	3%	1%	1.4	0.8
… I lost confidence in the hospital	88%	8%	3%	1%	0%	1.2	0.5
… I lost confidence in my orthopedic surgeon	94%	4%	1%	1%	0%	1.1	0.4
… I am afraid of a negative impact on my disease	23%	14%	21%	30%	12%	2.9	1.4
… I am more concerned about my disease	14%	18%	21%	26%	20%	3.2	1.3
… I have more disease associated limitations in my private life	26%	7%	12%	34%	21%	3.2	1.5
… I have more disease associated limitations in my occupational life	52%	11%	8%	13%	17%	2.3	1.6
… I am reconsidering the necessity of the surgery	78%	10%	1%	5%	5%	1.5	1.1
… I decided to even further postpone the surgery	58%	12%	12%	6%	11%	2.0	1.4
… I underwent surgery in another hospital	100%	0%	0%	0%	0%	1.0	0

Percentage of answers for each item and degree of agreement as well as mean point value with SD for each item were calculated

A mean PHQ-9 score of 6.1 ± 4.9 was found in the present cohort after cancellation of the scheduled surgery. Individual scores ranged from 0 to 23 points. A score of 10 points or above was found in 14 patients (18%). Six patients had a score of 15 points or more (8%). Subgroup analysis revealed that 5 out of 14 patients (36%) with a PHQ-9 score of 10 points or above were between 60 and 69 years old. Four of these five patients were female. In the 50–59 years age group, four patients (29%) with scores of more than 10 points were identified. Only two patients older than 70 years showed scores above the cut-off value. Two-way ANOVA revealed significantly higher PHQ-9 scores in female patients than in male patients, with 7.4 and 5.1 points, respectively (*p* = 0.046) (Table 2). No significant differences in PHQ-9 scores could be observed among age groups, procedures or reasons for cancellation.

## Discussion

The most important findings of the present study were a significant increase of pain level from the time of the initially scheduled surgery to the time of the survey. Additionally, significant psychosocial distress due to the cancellation was identified in some patients, particularly middle-aged women.

A newly developed questionnaire including the validated PHQ-9 was used to survey patients whose elective operations in the orthopaedic department had been postponed due to the COVID-19 pandemic. The response rate was 65% and is therefore in line with a large analysis by Mindell, who reported average response rates of 45–66% in seven large national health examination surveys in Europe, and with a meta-analysis by Baruch, who identified an average response rate of 48% in 490 survey studies [3, 25].

The gender ratio shows a predominance of males in contrast to the general ratio for endoprosthetic joint replacement in large study groups [15]. This is due to the relatively high proportion (25%) of hip arthroscopies in the patient population, in which male patients predominate. The risk of developing COVID-19 increases with age (> 50 years), and these patients are considered to be at risk [12, 22, 27, 35]. In the study cohort, 69% of the participating subjects can thus be classified as high-risk patients. For this reason, it is surprising that only 16% of the patients cancelled their surgery on their own due to fear of a COVID-19 infection as part of the hospital treatment. Interestingly, 61% of the patients would have undergone their surgery during the pandemic, even though they were at risk. This is in accordance with a study by Parvizi et al., although 85% of patients understood and agreed with public health measures to curb infections [5]. In the present study, the majority (79%) of elective orthopaedic operations were cancelled by our institution according to governmental restrictions and general international guidelines [16, 17, 24, 33]. It was surprising that only 3% of all patients cancelled their surgery to leave capacity for the treatment of COVID-19 patients. This is possibly an expression of selfish behaviour in medical care during the COVID-19 pandemic. In this context, Garbe et al. investigated the relationship between personality traits and egoistic stockpiling of toilet paper in an online survey in 22 countries [10]. The results showed that people who felt more threatened by COVID-19 stored more toilet paper. The authors interpreted their results as an expression of concerns regarding the loss of control in this group. One could interpret the results of the present study as an expression of high pain levels rather than selfish behaviour. However, not all orthopaedic conditions included in the study were associated with increased pain levels which potentially supports the conclusion by Garbe et al., at least in some cases [10].

Multiple factors influence pain caused by osteoarthritis, which in turn has a significant impact on social and psychological functioning [2, 6]. In our cohort, the pain level increased significantly while waiting for surgery, despite 38% of our patients reporting an increased consumption of analgesic medication during the waiting period for the operation. In this context, Gregori et al. reported in a large systematic review and meta-analysis about uncertainty regarding the effect of popular painkillers such as nonsteroidal anti-inflammatory drugs compared to placebo on personal pain levels [11]. These results highlight the significance of the effect of mental aspects on subjective pain perception. On the other hand, Wang et al. reported increasing pain and radiographic signs of progression of osteoarthritis during 1 year of conservative treatment, emphasizing the physical component of pain in severe osteoarthritis [34].

Contrary to popular belief, women often exhibit higher pain levels, particularly in musculoskeletal disorders [9]. This could be confirmed in the present study; however, significantly higher pain levels in female than in male patients could only be identified before the operation was cancelled. The lowest pain levels among the musculoskeletal disorders in our study were identified for benign tumours. This can possibly be explained by the potential functional impairment due to the tumour but often in the absence of pain in the actual sense.

Samsson et al. emphasized that expectations of procedures such as surgery are closely related to perceptions of outcome [32]. Fortunately, the present study showed no general loss of faith in the national healthcare system or in the attending physicians in our department due to the postponement of the operation. Significant limitations in private and occupational life were not only due to cancelled surgery but also in general due to lockdown measures in the context of the COVID-19 pandemic. In the present study, patients hardly reconsidered the necessity of the operation. The primary indication was even confirmed by persistent or increasing pain. In addition to pain level, many other factors, such as income and socioeconomic status, influence waiting time for elective surgery, independent of the current pandemic [26]. However, only a few studies have investigated the potential influence of waiting time for elective surgery on postoperative outcome. In this context, Nilsdotter et al. conducted a study including 124 patients who underwent total hip arthroplasty in a timely manner or with a delay of 3 months (which roughly corresponds to our waiting time in the context of the coronavirus pandemic) [29]. In their study, the authors found no difference in postoperative outcome after 1 year. Similar results were published by Quon et al. [31]. They examined the effect of a 3-month waiting time on pain intensity after electrosurgical lumbar discectomy and identified a modest likelihood of experiencing more pain 6 months postoperatively in the delayed surgery group.

As shown in Table 1, the severity of the depressive mood is divided into four levels using the PHQ-9 questionnaire. In the present study, 14% of the participants met the criteria for moderate or severe depression with more than 10 or 15 points, respectively. This is perfectly in line with a PHQ-9 validation study conducted by Martin et al., who identified a prevalence of depressive symptoms of 9.2% among 2066 subjects in a representative sample [23]. In another study including 5018 subjects, Kocalevent et al. found a prevalence of major depressive disorder of 5.6% [14]. Again, this is supportive of our results, as we identified 8% of patients with major depressive symptoms. Furthermore, in concordance with Martin et al. and Kocalevent et al., we observed significantly higher scores in females than in male patients (7.4 vs. 5.1 points; *p* = 0.0046). In contrast to Kocalevent et al., the highest PHQ-9 scores in our cohort were observed in middle-age individuals, not old individuals [14]. However, this could be explained by the fact that they examined a representative population sample compared to the patient group with an orthopaedic disease in our study. Considering the proportion of patients with moderate to severe depressive symptoms, psychological support would be desirable, but this also poses a particular challenge in terms of availability in times of the COVID-19 pandemic.

However, several limitations of the present study have to be noted. First, the survey was only conducted at a single time point after the surgery was cancelled during the COVID-19 pandemic. Thus, no change in psychosocial distress before and after the cancellation of the surgery could be identified. Second, the cohort consisted of patients with a wide variety of orthopaedic conditions that are accompanied by different functional limitations. Third, only the PHQ-9 was previously validated which is potentially limiting the interpretation of the results. Fourth, the relatively small number of patients in the study cohort must also be noted as a further limitation.

## Conclusions

Cancellation and postponement of elective orthopaedic surgery caused significantly higher pain levels than those at the time when the surgery was scheduled, potentially leading to increased analgesic consumption. Additionally, significant psychosocial distress due to the cancellation may be induced in some patients, particularly middle-aged women. Despite these results, confidence in the national health care system and in the treating orthopaedic surgeons was not affected. However, to date, it is not clear whether the postponement of the surgery will lead to an inferior functional outcome postoperatively.

## Supplementary Information

Below is the link to the electronic supplementary material.

Supplementary file1 (PDF 198 KB) Supp. 1 Newly developed questionnaire including items on sociodemographic aspects, type of scheduled surgery and reason for cancellation, restrictions in personal and occupational life due to the orthopedic disease and the PHQ-9.

## Abbreviations

COVID-19: Coronavirus disease 2019
THA: Total hip arthroplasty
TKA: Total knee arthroplasty
PHQ-9: Patient Health Questionnaire
SARS-CoV-2: Severe acute respiratory syndrome coronavirus 2
SF-36: Short Form-36
WHO: World Health Organization
VAS: Visual analogue scale
