# Supplementary material for: COVID-19-related cancellation of elective orthopaedic surgery caused increased pain and psychosocial distress levels
Source: Knee Surg Sports Traumatol Arthrosc. 2021 Mar 12;29(8):2379–85. doi: 10.1007/s00167-021-06529-4 (PMC7952835; doi:10.1007/s00167-021-06529-4)
Supplement: Supplementary file 1 — Supplementary file1 (PDF 198 KB) Supp. 1 Newly developed questionnaire including items on sociodemographic aspects, type of scheduled surgery and reason for cancellation, restrictions in personal and occupational life due to the orthopedic disease and the PHQ-9. [file 167_2021_6529_MOESM1_ESM.pdf]

Questionnaire on physical and psychosocial distress due to COVID-19 related postponement of elective orthopedic surgery

1. **Which kind of surgery was scheduled?**

- ☐ Total hip arthroplasty
- ☐ Revision total hip arthroplasty
- ☐ Partial or total knee arthroplasty
- ☐ Revision total knee arthroplasty
- ☐ Joint-preserving hip surgery (hip arthroscopy)
- ☐ Diagnostic arthroscopic biopsy of hip or knee prosthesis
- ☐ Foot or ankle surgery
- ☐ Resection of benign tumor

2. **How old are you?**

- ☐ < 30 years
- ☐ 30 - 50 years
- ☐ 50 - 60 years
- ☐ 60 - 70 years
- ☐ 70 - 80 years
- ☐ > 80 years

3. **What is your gender?**

- ☐ Female
- ☐ Male

4. **Who cancelled your scheduled surgery?**

- ☐ I cancelled the operation due to concerns of being infected with COVID-19
- ☐ I cancelled the operation to keep the clinic's resources available for COVID-19 patients
- ☐ The surgery was canceled by the clinic due to governmental ordinance during the pandemic

5. **Would you have undergone the scheduled surgery during the pandemic (if possible)?**

- ☐ Yes
- ☐ No
- ☐ Yes, if the health system would have had sufficient capacity for my surgery AND the treatment of COVID-19 patients

6. **Pain level BEFORE the pandemic at the time when the surgery was scheduled (0=no pain, 10= worst possible pain; please encircle correctly)**

0 – 1 – 2 – 3 – 4 – 5 – 6 – 7 – 8 – 9 – 10

7. **Pain level AFTER cancellation of the surgery due to the pandemic (current pain level) (0=no pain, 10= worst possible pain; please encircle correctly)**

0 – 1 – 2 – 3 – 4 – 5 – 6 – 7 – 8 – 9 – 10

8. **Subjective urgency of the scheduled surgery BEFORE the pandemic at the time when the surgery was scheduled (0 = the surgery is not urgent at all, 5 = the surgery is highly urgent; encircle correctly, please)**

1 – 2 – 3 – 4 – 5

9. **Subjective urgency of the scheduled surgery AFTER cancellation of the surgery due to the pandemic (current situation)**
(0=the surgery is not urgent at all, 5=the surgery is extremely urgent; please encircle appropriately)

1 – 2 – 3 – 4 – 5

Due to the postponement of my scheduled surgery, I have...

	Disagree (1)	Tend to disagree (2)	No impact (3)	Tend to agree (4)	Agree (5)
10. lost faith in the German health care system	☐	☐	☐	☐	☐
11. lost faith in my treating hospital	☐	☐	☐	☐	☐
12. lost faith in my treating orthopedic surgeon	☐	☐	☐	☐	☐
13. concerns regarding negative effects for my disease	☐	☐	☐	☐	☐
14. worried more about my illness	☐	☐	☐	☐	☐
15. experienced restrictions in private everyday life	☐	☐	☐	☐	☐
16. experienced restrictions in occupational everyday life	☐	☐	☐	☐	☐
17. reconsidered if the surgery is still necessary	☐	☐	☐	☐	☐
18. more pain	☐	☐	☐	☐	☐
19. taken more painkillers	☐	☐	☐	☐	☐
20. decided to postpone the surgery even further	☐	☐	☐	☐	☐
21. undergone the surgery elsewhere	☐	☐	☐	☐	☐

Over the last <u>2 weeks</u>, how often have you been bothered by any of the following problems? (PHQ-9)

	Not at all (0)	Several days (1)	More than half the days (2)	Nearly every day (3)
22. Little interest or pleasure in doing things	□	□	□	□
23. Feeling down, depressed or hopeless	□	□	□	□
24. Difficulty falling or staying asleep, or sleeping too much	□	□	□	□
25. Feeling tired or having little energy	□	□	□	□
26. Poor appetite or overeating	□	□	□	□
27. Feeling bad about yourself – or that you are a failure or have let yourself or your family down	□	□	□	□
28. Trouble concentrating on things, such as reading the newspaper or watching television	□	□	□	□
29. Moving or speaking so slowly that other people could have noticed. Or the opposite – being so figety or restless that you have been moving around a lot more than usual	□	□	□	□
30. Thoughts that you would be better off dead, or of hurting yourself	□	□	□	□
